# A Few Suggestions on the Prevention of Tuberculosis*Read before the North Carolina Medical Society, May, 1898.

**Published:** 1898-06

**Authors:** James A. Burroughs

**Affiliations:** Asheville, N. C.


### A FEW SUGGESTIONS ON THE PREVENTION OF TUBERCULOSIS.*

BY JAMES A. BURROUGHS, M.D., ASHEVILLE, N. C.

There has been so much written on the subject of tuberculosis since Professor Koch in 1882 first discovered the germ that causes consumption, we hesitate in opening any phase of the subject, yet we are excusable when we state, from statistics, that this is a disease that causes the death of every seventh human being on the globe, and further, if proper precautions and sanitary regulations were observed, in short time the number of cases could be greatly reduced and longevity raised several years.

All admit that tuberculosis is an infectious and contagious disease, and, at the same time, must admit that all contagious and infectious diseases could be prevented if we only knew what to do.

Located as I am, handling hundreds of these cases from every quarter of the United States and Canada, it is appalling to note the lack of knowledge of the contagiousness of tuberculosis and the great danger the infected are to others. It is no infrequent occurrence for a tubercular mother to expect to keep a baby and a few small children in her bedroom, as no protest has been entered against the practice by the family physician at home.

As soon as the diagnosis of tuberculosis is made, first of all, without an exception, it should be communicated to the patient, and he should be informed of all known facts on the contagiousness of the disease, so that he would not be a source of danger to others or reinfect himself. The sputa is the principal means by which contagion is spread. All the sputa should be collected in a sanitary hand cuspidor and the papers removed at least twice daily and exposed to a strong solution of bichloride of mercury, or cremated; see to the thorough destruction of these papers of sputa; do not allow them to be thrown into the sink to infect people or cattle miles away; do not permit a blaze to consume the dry portions of paper, leaving the sputa with its millions of germs unmolested to dry and do their deadly work; make the patient understand that it is the sputa you wish to have thoroughly burned.

Patients in the first stage, in fact all stages, of the disease do a greater portion of their coughing and expectorating on rising in the morning, and it incurs no hardship to demand this duty of them, in which they soon acquiesce.

About two years ago the little city of Asheville, N. C., passed an anti-expectoration ordinance (which was, so far as I know, the first ordinance of the kind in this country), since which time one hundred and seventeen cities and towns in the United

[*Read before the North Carolina Medical Society, May, 1898.

States have passed a similar law. It has proven a great educational feature with the masses, and practically put a stop to the dangerous insanitary nuisance of spitting upon the sidewalks, in the street-cars and public buildings, in the city where the anti-expectoration ordinance was originated. All cities that have passed this ordinance have experienced trouble in enforcing a strict letter of the law. Millionaires have instituted suits for damages against corporations because of reproof and ejectment for vulgarly violating this ordinance, but this has only had a good, wholesome, and stimulating effect upon the people; it has put them to thinking.

The poor consumptive of to-day, who is properly informed, is careful with his sputa; he feels that indifference on this point may be death to others, cause him litigation, or hasten a crisis in his own case.

It is an admitted fact that no children are born with tuberculosis, yet they do inherit that peculiar lymphatic, anemic, poorly nourished constitution which gives them but little or no resistance when exposed. To permit these unfortunate children to reside in tubercular homes, with like habits and environments as their infected parents or relatives, is nothing short of criminal negligence. Such children should be removed to an altitude of dry, rarefied atmosphere loaded with ozone and perpetual sunshine. In conducting them into puberty and settled life, short school hours should be provided, with much time spent in the open air. This class should be induced and taught the necessity of eating such food as will make blood and build up good, healthy tissue cells, so as to give them all possible means of resisting any invasion of this dreaded enemy.

I have at this time under my supervision many individuals and families of a tubercular diathesis who have undoubtedly escaped the disease by the above suggestions. At this moment I call to mind three younger members of a New Jersey family of ten, all of whom had died in quick succession with tuberculosis. These three located in Western North Carolina eight years ago, and no symptoms of tuberculosis have developed in either of them; the younger is now two years older than any one of the brothers or sisters who died in New Jersey.

A complete change of climate in all tubercular families is wise sanitary advice, and, to repeat, that change should be to a higher altitude where the air contains a quantity of ozone and perpetual sunshine, and is freer from gases and bacteria. It is judicious to note here that ozone and sunshine are deadly to tubercular bacilli, and to this fact is due immunization of the points like the City of Mexico, Denver and Asheville, that have been for years resorts for consumptives.

It is possible for the well-to-do class to avail themselves of these sanitary precautions, but it is quite different with those less fortunate. What we shall do with our tubercular poor is a big question, and one which demands the most serious and careful consideration by every State and municipal board of

Health. To diagnose these cases and report them to the local boards and have them registered, does but little good beyond the general advice given, and swelling our statistics. What that class needs is some provision by State or city where they could be placed under the best sanitary regulations, so as to protect an innocent community, and, at the same time, offer them the best chance for an arrest or cure. It is more necessary that this class should have official care, as they are largely ignorant and careless, and it is principally these people who lounge around lawns and suburban fields scattering millions of germs upon the grass which infect fowls, stock and milk.

England has greatly reduced her death rate of pulmonary tuberculosis by providing hospitals for her poor. England has eighteen tubercular hospitals, with the capacity of seven thousand beds.

With the exception of the States of New York and Massachusetts, there has been no attempt at isolation of the bulk and file of tuberculosis in this country, so far as I know.

It is gratifying to note the good being accomplished in veterinary surgery under the various State boards of health in pointing out infected herds of cattle and disposing of same.

Every large collection of people should have a competent bacteriologist to keep a watch over its meat and milk supply, with an especial view to prevent tubercular infection. This official would doubtless save the corporation employing him many times his salary, in pauper burial expenses, to say nothing of the protection to human life.

Malnutrition, insufficient food of a poor quality, and overcrowding of tenements and public buildings are subjects that are constantly confronting health boards and individual practitioners; each is a subject within itself.

Wish to say something in behalf of our overcrowded school buildings and orphan homes. A glance at the pale anemic faces in a public schoolroom would convince the most skeptical of the vicious results of improper ventilation. That many children become infected in the vitiated atmosphere of the schoolroom is a painful fact that is only too often brought to our notice.

If mountain air, containing, as it does, large quantities of ozone, is beneficial, and often curative, in tuberculosis, why not bring a similar condition to bear in the insanitary schoolroom or other places where there is assembled a large and permanent collection of people?

It is a somewhat remarkable fact that ozone, which can be generated by means of an electric current with comparative economy and cheapness, has not been put to more extensive use for sanitary and prophylactic purposes. The extraordinary germicidal power of ozone has long been known and recognized by the scientific medical world; the ozonizing of the schoolroom and public building is a matter of minor mechanics. An ozone-generating apparatus is not expensive and can be connected with an electric current from street-car wire coming into the building, or with a private dynamo; with the proper apparatus the whole building can be impregnated with this germ-destroying gas.

In any large assemblage of children some one or more have tuberculosis which has not been suspected by teacher, family, or consulting physician. If the ozonizing of school buildings were put into practice, that source of tuberculosis, with all other contagious and infectious diseases, would be practically cut off, and the children would have a nice, sweet, clean, non-germ-laden fluid to breathe. This idea adopted in the school, there would be fewer constitutions prepared for tubercular infection.

In my judgment, no hospital or sanatorium, either private or public, should be kept open unless there was ample provision for ozonizing the entire building from cellar to garret. The statistics of mortality from tuberculosis in insane asylums and prisons of the different States, is sufficient to suggest a consideration of the above idea; all must acknowledge that something is wrong when strong men in a few months after incarceration become tubercular. With an ample ozonizing apparatus placed in these prisons and asylums, there would be marked diminution of tuberculosis.

The ozonizing of all hotels, and especially Pullman cars, is the most rational solution of the danger along that line. Permit me to state that in one of the "babies wards" of the New York Post-Graduate Hospital, where an ozone-generating apparatus is used, contagious diseases are almost a curiosity, whereas in non-ozoned wards quite a number have occurred.

Some ingenious pathologist has stated that the consumptive cadaver contains about eleven million tubercle bacilli. I am not prepared to substantiate or deny the above statement, yet it is quite reasonable to presume that the calculation is approximately correct. We do know that these bacilli will live in the ground for more than a quarter of a century, retaining all the vitality of the same germ that was carelessly deposited in a hotel lobby the day before. Knowing this, it does seem reasonable and practicable, from a prophylactic and sanitary position, that all tubercular dead should be cremated; to bury this class means to infect the soil and contaminate dependent water supplies for man and beast for more than a generation. A concerted action of all the civilized world in cremating its tubercular dead might not have much effect upon this generation, but would lessen the dreaded disease for those who come after us.

There are many points of vital value on the prevention of tuberculosis left out of this little paper which I trust may be brought out in the discussion.

The laity, as well as the profession, is waking up to the contagiousness of this disease, and, in the judgment of some of our best thinkers, there will soon be a pronounced recognizable reaction in the number of the tubercular; indeed a perceptible reaction has already begun, as is observed from an editorial in the Journal of the American Medical Association of February 26th, 1898, where the statistics from twenty of our principal cities, having a population of seven million and five hundred thousand, have shown a decrease of the tubercular death rate of thirty-three per cent. since 1888, which is tersely ascribed to a more general knowledge of the contagiousness of the disease, better food-supply and more perfect sanitation.

DISCUSSION.

DR. A. A. KENT: I feel like adding more than simple emphasis to the position taken by Dr. Burroughs, for I do wish, gentlemen, to emphasize the importance of preventative measures in tuberculosis. If tuberculosis is a contagious, infectious disease, then it is preventive, and if preventive, it behooves us, as custodians of the public health, to bestir ourselves that something effective may be done. Dr. Koch, some sixteen years ago, demonstrated the fact of the causal relation of the bacillus of tuberculosis to consumption. During these sixteen years of time the most able scientists of the world have been confirming his conclusions. There are some who still doubt, that we must admit, but while we admit it I am frank to say that there being some who have doubts tends to make discoverers more careful, more painstaking in promulgating their discoveries. It stimulates research, but the fact that there are those who doubt does not disprove it, and it stands to-day among the best of the profession a well-admitted fact that consumption is a contagious disease. Then, if contagious, what must we do? Shall we ask the legislature to pass laws? In my opinion we must, but before we can enforce laws we must have the moral support of the public, of the people; we must teach it in season and out of season; we must so teach it and preach it that the people will learn it, and that once having learned it they may put it into practice, for no knowledge is of value that can not be put into practical use. I am glad to be able to say that our highest institutions of learning are already armed and equipped with the proper information on the subject. I have had some little correspondence with our best-equipped institutions, and I find that they are already enforcing proper sanitary regulations in regard to this disease, and I am glad that it is a fact. These are the fountains, the sources of knowledge, and from them it will be spread abroad among the people. But, gentlemen, more may be done, and more may be done quickly. It should be taught in the homes; it should be taught in the common schools; it should be taught in the high schools; it should be taught in the colleges; it should be taught in the hotels and boarding houses; it should be taught in the church and in the State; and we should continue to so teach and so preach it that the people will be ready to join hands with us in the battle against this arch-enemy of human life. If we once havethe people educated on the subject, we are ready for strict laws and the laws will be enforced. But, gentlemen, it behooves us to teach the people, and when once we have taught them, with willing hands, educated brains and determined minds they will take up the work with us, and in one common cause we can go on to the extermination of this the greatest enemy of human life.

DR. LEWIS: I want to express my gratification at the excellent paper read by Dr, Burroughs, and the no less excellent remarks by Dr. Kent, and I think he sounds the keynote when he speaks of the profession as the custodians of the public health. I don't think anybody can deny that every physician is a health officer morally, whether he is so really or not. It is simply a matter of education. Laws are nothing unless the people are willing to carry them out. There are one or two practical things that I think all of us could carry out without any trouble at all if we would simply give it attention. The ordinance against spitting on the streets of a city or town is very well, and I am mighty glad the laws of our city of Asheville have done that, but you all know, as stated by Dr. Burroughs, that sunshine and ozone are fatal to the life of the tuberculosis bacilli. People spitting on the streets, out in the open air, is not so dangerous as their spitting in the unventilated houses where these bacilli flourish. As illustrating this point, I quote from the last bulletin of the State of Ohio, which is just out, the history of a house in Ohio in which there had been three deaths, evidently contracted from an original case in it. It was a new family who moved into the house, and who had no hereditary predisposition to the disease. Now that is a striking object-lesson, and I am satisfied bears very materially on the question. If the profession insists upon it, and impresses upon their patients the fact that tuberculosis is a contagious disease, and unless they do take the proper precautions they not only cause their own death but the death of those dearer to them than their own lives, I think there would be no difficulty in carrying out the proper precautions. As I intimated, the most important of all is the thorough disinfection of rooms that have been occupied by tuberculosis patients. If we could ever get the sentiment instilled into the minds of the patients in the towns and cities that they would under no circumstances rent a house in which a case of consumption had lived unless that house had been thoroughly disinfected, and an official certificate to that effect presented, I believe it would have a very great effect. If the profession could succeed in poisoning the minds of the patients against the landlords to that extent, and impress upon their patients that in no case should they occupy a house unless that house had been disinfected, I believe public opinion would be built up more rapidly than in any other way I know of. Of course, isolation of tuberculosis patients is desirable. Practically, of course, it can not be done. You can't take the children away from their parents. The poor you can't manage because they haven't got the money, and as we all know, public sentiment in North Carolina is not ready to make appropriations to take care of consumptives. If the profession can impress upon their patients the fact that tuberculosis is a contagious disease, and above all that absolutely thorough disinfection and ventilation of rooms occupied is necessary, which can be most easily done, I do believe a great deal of good may be accomplished.

DR. J. W. LONG: I would like to say a few words in discussing this paper, but it so fully covers the ground that it has left very little to be said, and I rise simply to endorse what has been said. I think that we cannot speak upon the prevention of tuberculosis too often. Every gentleman who has spoken has emphasized the necessity of educating the public along this line, and the only way we can educate the public is to keep talking about it, and I do not believe we can spend an hour more profitably in any medical meeting than in discussing this question of the prevention and the means of preventing tuberculosis. There are only one or two points that I would call attention to, and one is the practical matter of preventing tuberculosis among physicians themselves. I think possibly, if you will excuse the personal reference, that I inherit a tuberculous tendency from my father, and every time I go to see a tuberculous patient it hangs over me like a terrible nightmare, and I take the precaution after every visit, whenever it is possible for me to do so, to wash my hands and face, because it is by these means that the bacilli are conveyed to the lungs. Now, I think that the very fact that tuberculosis is so mildly infectious constitutes the dangerous element. If we could show to the public that tuberculosis was acutely infectious, as is scarlatina or diphtheria, we might hope in a short time to educate them up to seeing the necessity of instituting measures to prevent it, but it is because it is so mildly infectious that we have such a hard time in educating the public. But returning to the point which I just mentioned, and that is tuberculosis among physicians. We all of us meet doctors occasionally who have tuberculosis—we know of doctors who have died of tuberculosis. The question comes up, how did they contract it? And it is quite possible that they contracted it through contact with tuberculous patients, and I think we might set a good example to the laity by instituting some reform ourselves, and that we should show them that it is important to disinfect ourselves. Now, I have heard physicians talk about disinfection after visiting cases of scarlatina, and yet those very same doctors would go to a case of consumption and never disinfect their hands, or their faces, or their overcoats, and go to another patient (a child it may be) without any disinfection. We can best educate the public by instituting some reform ourselves along this line. I was very much pleased with Dr. Burroughs' paper. It kind of knocked me out on one thing, though. He said that every sanitarium ought to have an ozonizing apparatus, and I kind of consoled myself by thinking that this might be necessary for the sanitariums in New York or Asheville, but I didn't think it was nec essary for the sanitarium at Salisbury. It may be needed in Asheville, the great Mecca for consumptives, but we don't want you to use any ozonizing apparatus on us. I was very much pleased with Dr. Burroughs' paper and feel very much profited by the discussion of it.

DR. LEWIS: Dr. Spencer says that the keeper of the poorhouse or home for the aged and infirm of Caswell county died not quite two years ago of phthisis. No family history whatever. Since that time two children have died. Two remaining children have now a phthisis. The wife has tuberculosis, and two laborers that spent a week in that house have tuberculosis. I asked his advice in regard to the disinfecting of that house, and he gave the advice that the best way was to burn it up. That is really a very striking lesson.

